# A Mutant Library Approach to Identify Improved Meningococcal Factor H Binding Protein Vaccine Antigens

**DOI:** 10.1371/journal.pone.0128185

**Published:** 2015-06-09

**Authors:** Monica Konar, Raffaella Rossi, Helen Walter, Rolando Pajon, Peter T. Beernink

**Affiliations:** 1 Center for Immunobiology and Vaccine Development, Children’s Hospital Oakland Research Institute, UCSF Benioff Children’s Hospital Oakland, Oakland, CA, United States of America; 2 Biology Department, Mills College, Oakland, CA, United States of America; Cornell University, UNITED STATES

## Abstract

Factor H binding protein (FHbp) is a virulence factor used by meningococci to evade the host complement system. FHbp elicits bactericidal antibodies in humans and is part of two recently licensed vaccines. Using human complement Factor H (FH) transgenic mice, we previously showed that binding of FH decreased the protective antibody responses to FHbp vaccination. Therefore, in the present study we devised a library-based method to identify mutant FHbp antigens with very low binding of FH. Using an FHbp sequence variant in one of the two licensed vaccines, we displayed an error-prone PCR mutant FHbp library on the surface of *Escherichia coli*. We used fluorescence-activated cell sorting to isolate FHbp mutants with very low binding of human FH and preserved binding of control anti-FHbp monoclonal antibodies. We sequenced the gene encoding FHbp from selected clones and introduced the mutations into a soluble FHbp construct. Using this approach, we identified several new mutant FHbp vaccine antigens that had very low binding of FH as measured by ELISA and surface plasmon resonance. The new mutant FHbp antigens elicited protective antibody responses in human FH transgenic mice that were up to 20-fold higher than those elicited by the wild-type FHbp antigen. This approach offers the potential to discover mutant antigens that might not be predictable even with protein structural information and potentially can be applied to other microbial vaccine antigens that bind host proteins.

## Introduction

Binding of complement regulators is a common strategy that microbes use to subvert the host immune system. It has been proposed, therefore, that vaccines targeting the microbial ligands for host complement regulators would elicit antibodies that defeat this evasion mechanism [1]. However, the effectiveness of microbial proteins that bind host complement regulators as vaccine antigens might be hampered by binding of the host protein. Hence, modification of the microbial antigen so as not to bind the complement regulator might increase immunogenicity [1]. Several microbial vaccine antigens that bind complement regulators, including HIV gp120 [2] and Neisserial surface protein A (NspA) [3], have been tested in human clinical studies. Although structures of these antigens have been determined experimentally [4, 5], the approach of modifying the vaccine antigen so it no longer binds the host molecule has not yet advanced to testing in humans.

*Neisseria meningitidis* (meningococcus) causes cases of bacterial meningitis and sepsis [6], which can be fatal or result in permanent damage. The major meningococcal ligand for the complement regulator Factor H (FH) is Factor H binding protein (FHbp), which is an important component of two recently licensed vaccines against serogroup B meningococci [7, 8]. The open reading frame encoding FHbp initially was identified as NMB1870 from the genome sequence of strain MC58 [9]. The protein was renamed FHbp after it was discovered to bind the human complement regulator Factor H (FH) [10]. Subsequent studies showed that binding of FH to FHbp was specific for human and chimpanzee FH [11] and for FH from a subset of rhesus macaques [12]. Vaccines containing FHbp are immunogenic in mice [13, 14], rabbits [15], macaques [16, 17] and humans [7, 8, 18–20]; however the protective antibody responses of humans appear to be lower than those of mice [21].

Using human FH transgenic mice, we previously showed that binding of human FH decreased meningococcal FHbp vaccine immunogenicity compared with that in control mice [22]. The structure of a complex of FHbp bound to a fragment of human FH enabled the design of FH non-binding FHbp mutants [23]. We subsequently identified an FHbp mutant with arginine 41 replaced with serine (R41S), which retained immunogenicity in control mice in which FH does not bind FHbp, and had enhanced immunogenicity in human FH transgenic mice [22, 24]. Our hypothesis is that a mutant FHbp antigen with very low FH binding and increased immunogenicity in human FH transgenic mice will translate into greater vaccine efficacy in humans. Many FHbp mutants with decreased binding of human FH since have been identified [25–28]. However, some FHbp mutants have decreased immunogenicity compared with a wild-type FHbp vaccine in control mice in which FH does not bind to either FHbp vaccine. These data suggest that mutations that decrease FH binding can result in the loss of epitopes important for eliciting protective antibody.

In the present study, we developed a novel approach to identify novel FHbp antigens using a random mutant FHbp library displayed on the surface of *Escherichia coli*. This approach has the advantages of not relying on protein structural information and the potential to identify various amino acid substitutions at many different positions. We used this approach to identify several new mutant FHbp antigens that exhibit very low binding of human FH and that have the potential to be more effective in humans compared with FHbp antigens that bind FH.

## Materials and Methods

### Construction of FHbp mutant library

Using the Diversify error-prone PCR mutagenesis kit (Clontech), we amplified the FHbp ID 1 (http://pubmlst.org/neisseria/fHbp) gene, including the native signal sequence, to introduce approximately four substitutions per 1000 bp. Based on the size of the FHbp gene (822 bp) and the degeneracy of the genetic code, we predicted an average of two amino acid substitutions per FHbp molecule. The error-prone PCR products were incubated with *Taq* DNA polymerase and dNTPs at 70°C for 10 min to introduce dA overhangs, and were cloned into pGEM-T-Easy (Promega). The clones were transformed into *E*. *coli* DH5α (Invitrogen). Plasmid DNA was prepared (Qiagen) and 30 clones were subjected to DNA sequencing with the T7 primer. The mutation rate was 3 to 5 nucleotides per FHbp gene, and one to two amino acid residues per FHbp. The error-prone mutant library was digested with the restriction endonucleases *Nco*I and *Xho*I and cloned into plasmid pET28a (Novagen). The ligation products were transformed into electrocompetent *E*. *coli* C41(DE3) (Lucigen) for surface expression of the FHbp mutant library.

### Fluorescence-activated cell sorting of *E*. *coli* display library

For sorting of *E*. *coli* cells expressing mutant FHbp, the library was grown in Luria-Bertani (LB) medium (BD Biosciences) containing 50 μg/ml of kanamycin (Sigma) to stationary phase. The cultures were diluted 1:25 into fresh LB medium and grown to an OD at 600 nm of 0.6. FHbp expression was induced with 1 mM IPTG for 2 h at 37 °C. The bacterial cells were incubated with 50 μg/ml of purified human FH (Complement Technologies) for 30 min at 37°C. The cells were washed twice with Dulbecco’s PBS (DPBS) (Mediatech) containing 1% BSA (Equitech). The primary antibodies were goat anti-human FH (Complement Technologies; 2 μg/ml), which had been affinity purified on an FH column, or a mixture of control anti-FHbp mAbs JAR 41 [29] and mAb 502 [30] (10 μg/ml each), which were added and incubated for 30 min at 37°C. Note that neither of these mAbs inhibited binding of FH to FHbp [31], which enabled co-immunostaining with FH. The secondary antibodies were donkey anti-goat IgG conjugated to AlexaFluor 488 (Invitrogen; 1:1000 dilution) and donkey anti-mouse IgG-AlexaFluor 647 (Invitrogen; 1:1000 dilution), which were added and incubated for 15 min at room temperature. The cells were washed twice with DPBS and were suspended in 1 ml of PBS for sorting (FACSAria; BD Biosciences). The gating strategy used *E*. *coli* expressing wild-type FHbp as a positive control for FH and mAb binding and a similar FHbp clone encoding the R41S mutant [22] as a negative control for FH binding.

### Preparation of selected FHbp mutants

The sorted clones were grown overnight on LB agar plates containing 50 μg/ml of kanamycin. Single colonies were used as templates for PCR amplification of FHbp mutants using T7 and T7 terminator primers, and DNA sequencing was performed with the same primers. In the sorted clones, there was an average of two amino acid substitutions per FHbp (range of 1 to 4). To prioritize which substitutions to test further, we examined their locations in the structure of the FH-FHbp complex [23], and selected FHbp residues with polar side chains that were ≤5 Å from the nearest non-hydrogen atom in FH. Single amino acid mutants of soluble FHbp (i.e. lacking the signal sequence) were constructed using the Phusion Site-Directed Mutagenesis Kit (Thermo Scientific) and the primers listed in Table 1. The mutants were expressed from pET21b (Novagen) with a carboxyl-terminal hexahistidine tag [13] in *E*. *coli* BL21(DE3). The FHbp mutants were purified using immobilized metal afﬁnity chromatography and ion exchange chromatography [32].

**Table 1:** Primers used for site-specific mutagenesis.

Name^a^	Sequence^b^	Annealing Temperature (°C)^c^
Q38R_F	TCTTTGACGCTGGATCGGTCCGTC	60
Q38R_R	CTGCAAACCTTTGTCTTTATGGTCGAGCGG	60
E92K_F	ATTACCTTGAAGAGTGGAGAGTTCCAAACAA	65
E92K_R	GAGCTGCCCGTCCACTTCGATTTG	65
I114T_F	CAGACCGAGCAAACACAAGATTCGGAGCATTCC	70
I114T_R	AAAGGCGGTTAAGGCGGAATGGCT	70
R130G_F	AAACGCCAGTTCGGAATCGGCGAC	70
R130G_R	CGCAACCATCTTCCCGGAATGCTC	70
I134V_F	ATCGGCGACGTAGCGGGCGAACATACA	70
I134V_R	TCTGAACTGGCGTTTCGCAACCATCTTCCC	70
H138L_F	GCGGGCGAACTTACATCTTTTGACAAG	60
H138L_R	TATGTCGCCGATTCTGAACTGGCGTTT	60
S211T_F	ATCAGCGGTACCGTCCTTTACAACCAA	70
S211T_R	GACGGCATGGCGTTTTCCATCC	70
G236C_F	GAAGTTGCCTGCAGCGCGGAAGTG	70
G236C_R	CTGGGCTTTTCCGCCAAAGATACCGAG	70
H248L_F	ATACGCCTTATCGGCCTTGCC	65
H248L_R	GCCGTTTACGGTTTTCACTTCCG	65
S223R_F	AGTTACCGCCTCGGTATCTTTGGCG	60
S223R_R	GCCTTTCTCGGCTTGGTTGTAA	60
K199E_F	GCCGATATCGAGCCGGATGGAAAA	65
K199E_R	GGCGGCCAGGTCGACATTG	65

^a^ Name of primer for each mutant based on numbering of mature FHbp ID 1 sequence, beginning with lipidated cysteine residue; F, forward; R, reverse

^b^ Sequence shown from 5’ to 3’; nucleotide changes relative to wild-type sequence in forward primer are underlined

^c^ Annealing temperature used to amplify mutant FHbp gene

### Binding of FH to recombinant FHbp and evaluation of conformational integrity

Binding of FH to FHbp was measured in a modified ELISA [22] using as much as 200 μg/ml of purified human FH. Surface plasmon resonance experiments were performed by immobilizing human FH (Complement Technology; 1500 response units) on a CM5 chip (GE Life Sciences) via amine coupling. Soluble FHbp binding to immobilized FH was measured at concentrations ranging from 3.16 nM to 316 nM using a BIAcore X-100 Plus instrument (GE Life Sciences). Binding of control murine anti-FHbp mAbs to FHbp was measured by ELISA [33]. We measured the thermal stability of FHbp mutants by differential scanning calorimetry using a VP-DSC instrument (MicroCal), a protein concentration of 0.5 mg/ml and PBS as the diluent and reference solution. The data were collected using a scan rate of 60°C per hour with passive feedback mode and were analyzed using Origin 7.0 Software (MicroCal).

### Serum bactericidal antibody responses of mice

The experiments in mice were performed in strict accordance with the recommendations in the U.S. Department of Health and Human Services Guide for the Care and Use of Laboratory Animals. Following vaccination, blood was collected by cardiac puncture under isoflurane anesthesia and the animals were euthanized by cervical dislocation. The protocol was approved by the Institutional Animal Care and Use Committee of Children’s Hospital Oakland Research Institute. To determine whether epitopes important for eliciting bactericidal antibodies were preserved in the mutant FHbp, groups of four-week old female CD-1 mice (N = 14 per group) were given two i.p. doses, each containing 10 μg of FHbp adsorbed to 600 μg Alhydrogel (Brenntag Biosector) in 10 mM Histidine, 150 mM NaCl, pH 6.5. The vaccines were administered at three-week intervals and blood was obtained three weeks after the second dose.

To determine whether the mutant FHbp antigens with decreased binding of human FH gave greater protection than a control FHbp antigen that bound FH, we also conducted immunogenicity studies in human FH transgenic BALB/c mice. We first measured the human FH concentrations in the sera of the transgenic mice obtained prior to immunization using a FHbp capture ELISA [22] and only included mice with serum human FH concentrations >250 μg/ml. For the transgenic mouse immunogenicity study, we confirmed that the vaccine groups had similar mean serum human FH concentrations, ages and gender distributions. Groups of 6- to 12-week old mice (N = 15 per group) were immunized with three i.p. doses of FHbp (adsorbed to Alhydrogel as described above) at three-week intervals and blood was obtained three weeks after the third dose.

Human serum for use in bactericidal assays was obtained from a healthy donor with written informed consent under a protocol approved by the Institutional Review Board of Children’s Hospital & Research Center Oakland. Complement-mediated serum bactericidal antibody responses were measured using exponential-phase bacteria and human serum depleted of IgG as a complement source as previously described [34]. Human serum was obtained from a healthy donor who lacked bactericidal antibody against meningococci. SBA responses to mutant FHbp ID 1 vaccines were determined using serogroup B strain H44/76 (serologic classification B:15:P1.7,16; ST-32) [35, 36]. This strain is a high expresser of FHbp ID 1 [37], which matches the control wild-type FHbp antigen used for immunogenicity studies.

### Statistical analyses

SBA responses of groups of mice were compared using unpaired, non-parametric (Mann-Whitney) tests. The exact, two-tailed P values, as calculated in Prism 6.0e (GraphPad), are reported.

## Results

### Expression of FHbp on the surface of *E*. *coli* and sorting of an FHbp mutant library

**Fig 1:**
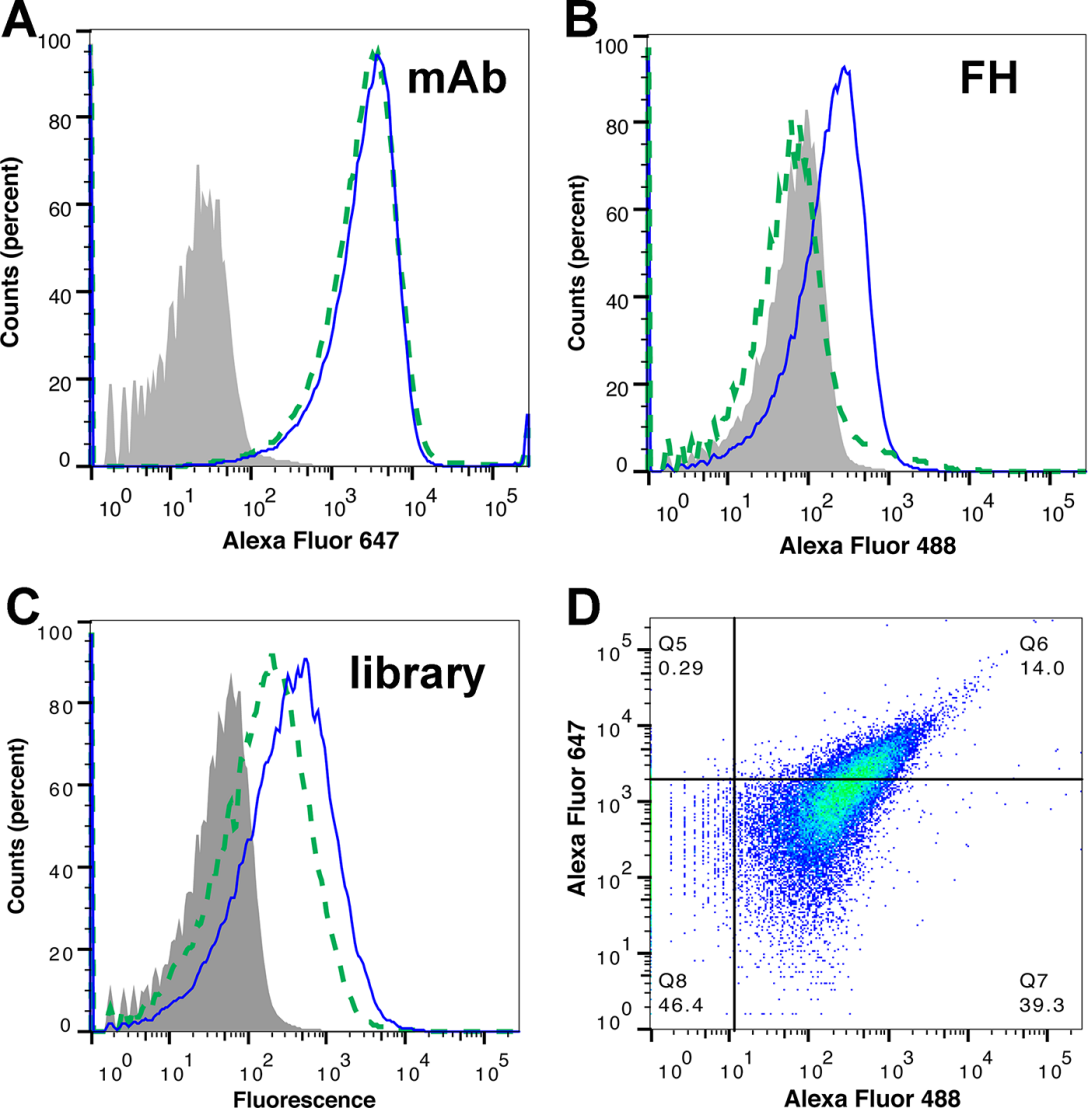
Detection of meningococcal FHbp on the surface of *E*. *coli*. **A.** Binding of anti-FHbp mAbs JAR 41 [29] and mAb502 [30] to *E*. *coli* transformed with plasmid expressing wild-type FHbp (solid blue line), R41S mutant (dashed green line) or empty plasmid (shaded grey histogram). **B.** Binding of purified human FH to same strains. **C.** Binding of *E*. *coli* expressing FHbp mutant library by the same mAbs as in Panel A (solid blue line) or human FH (dashed green line). **D.** Two-dimensional plot of FHbp mutant library stained simultaneously for binding of FH (Alexa Fluor 488) and anti-FHbp mAbs (Alexa Fluor 647). The FHbp R41S mutant (dashed green lines in Panels A and B) was used to define gates for the sub-population with high mAb binding and low FH binding (quadrant Q5 in upper left of Panel D; 0.3% of events).

Since binding of human FH decreases FHbp vaccine immunogenicity [22, 38], the objective of the present study was to identify new mutant FHbp antigens with very low FH binding. Therefore, we devised an *E*. *coli* display library and cell sorting approach to isolate such FHbp mutants. Meningococcal FHbp expressed with its native signal sequence was detected on the surface of *E*. *coli* by flow cytometry using a mixture of two murine anti-FHbp monoclonal antibodies (mAbs), JAR 41 [29] and mAb502 [30] (Fig 1A, solid blue line). The surface-expressed FHbp also bound human FH (Fig 1B, solid blue line). In contrast, the FHbp R41S mutant, which previously was shown to have >100-fold decreased binding of human FH [22], showed no significant binding of FH (dashed green line), similar to the negative control bacteria (shaded grey histogram). Although the R41S mutant did not bind FH, it was present on the bacterial surface since it was detected by the control murine mAb (**Fig 1A,** dashed green line).

Similar experiments were performed on *E*. *coli* expressing a random mutant FHbp library. The cells in the library bound the control anti-FHbp mAbs (Fig 1C, solid blue line). The *E*. *coli* mutant library also bound human FH (dashed green line), presumably because the majority of FHbp mutants in the library did not decrease binding of FH. Fluorescence-activated cell sorting (FACS) was performed by co-immunostaining with FH and anti-FHbp mAbs, neither of which inhibit binding of FH to FHbp [31], and the appropriate secondary antibodies (**Fig 1D**). Cells in the top left quadrant (0.3%) with high binding to the control mAbs and low FH binding were collected.

### Identification of FHbp mutants with decreased binding of human FH

**Fig 2:**
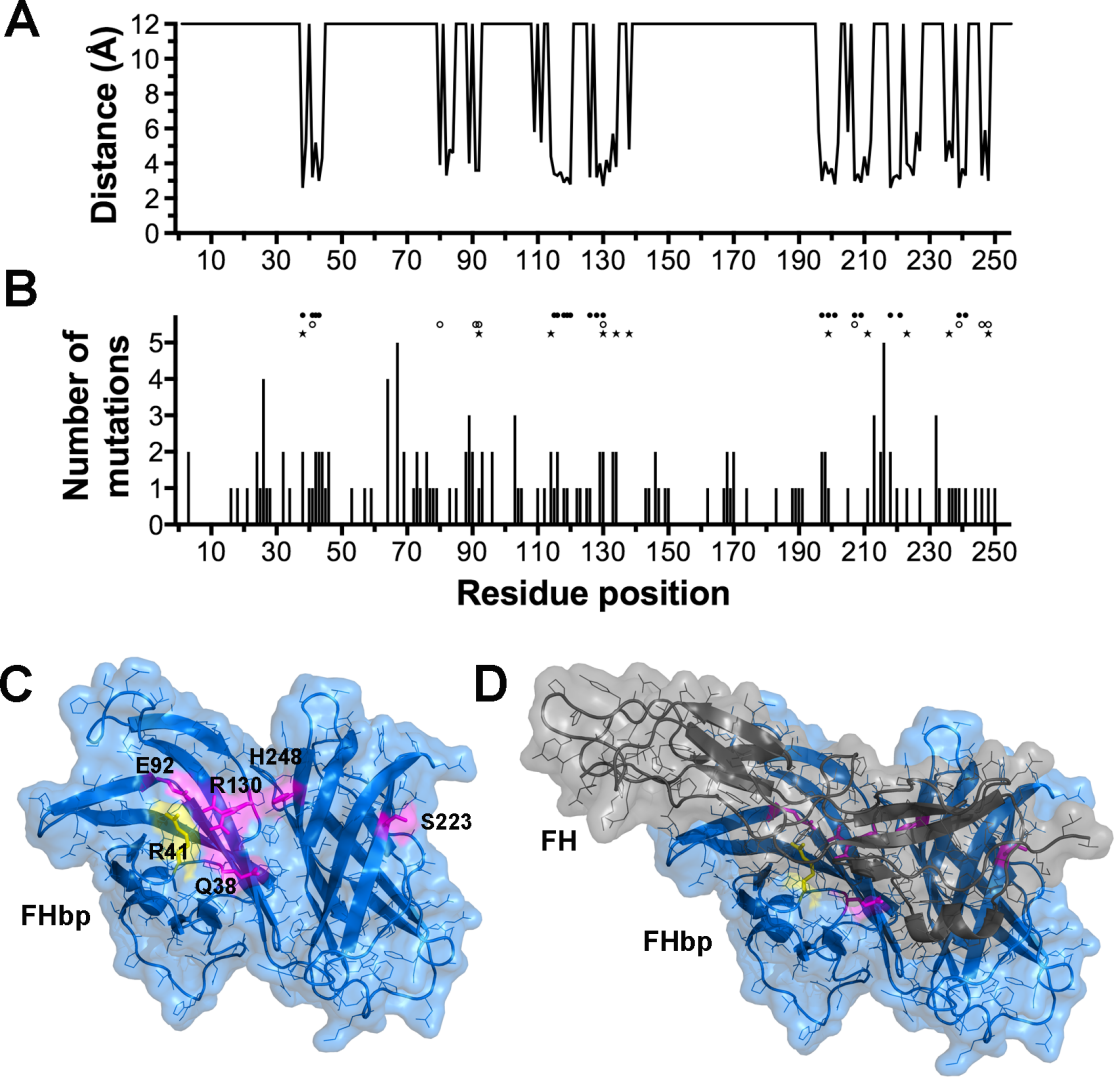
Location of mutations in FHbp in relation to FH. **A.** Plot of distance between each FHbp residue and closest atom in FH in the crystal structure of the FHbp-FH complex (PDB ID 2W80) [23]. Distances greater than 6 Å are depicted as 12 Å. The lipidated Cys residue in the mature protein is designated as residue number 1. **B.** Histogram showing number of mutations at each residue position in FHbp. Above the histogram are the known FH interaction residues (filled circles) [23], residues mutated in previous studies (open circles) [22, 23, 27] and residues mutated in the present study (filled stars). **C.** Location of residues with mutations identified to decrease binding of FH to FHbp (magenta). The location of residue R41, which previously was identified as an important FH binding residue is shown in yellow. **D.** Location of FH fragment (grey) relative to residues with mutations identified to decrease binding of FH to FHbp.

The *fhbp* genes from 120 clones were subjected to DNA sequence determination. To relate the positions of the FHbp mutations to the FH binding site, we mapped the distance between each FHbp residue and FH (Fig 2A). We then calculated the number of mutations observed at each FHbp amino acid residue position (Fig 2B). For reference, the positions of residues known to interact with FH [23] and residues previously investigated are shown [22, 23, 27]. Eleven amino acid positions were chosen for mutagenesis based on the solvent accessibility of the residues in the crystal structure of FHbp [39] and their proximity (<5 Å) to amino acid residues in FH based on the structure of FHbp bound to FH domains 6 or 7 [23]. Single amino acid substitutions were introduced into a soluble recombinant FHbp expression plasmid by site-specific mutagenesis. Of the eleven purified FHbp mutants, six showed little or moderate decreases (<10-fold) in binding of human FH compared with the wild-type FHbp, whereas five mutants had at least a 50-fold decrease in binding (Table 1). The locations of the latter five residues identified through the library approach are shown in the structure of FHbp in a complex with a fragment of human FH [23] (Fig 2C and 2D).

**Fig 3:**
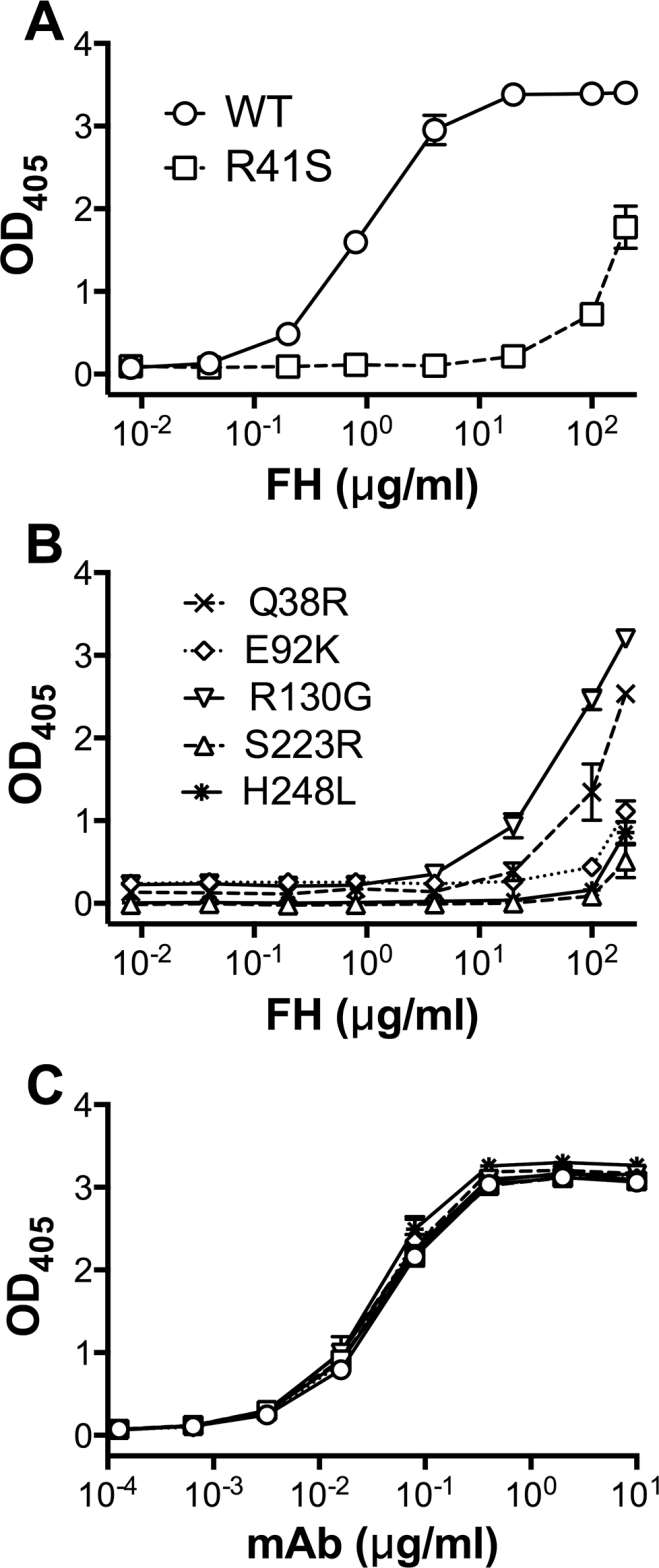
Binding of human FH and a control anti-FHbp mAb to FHbp mutants by ELISA. **A.** FHbp wild type (WT) bound human FH, whereas the R41S mutant had decreased binding. **B.** Binding of FH to the five mutants identified from display library approach. **C.** Binding of mAb JAR 5, which recognizes an epitope in the N-terminal domain of FHbp [40, 41], to WT, R41S and the five library mutants. In each panel, the mean and range of duplicate measurements are shown.

We determined the relative binding of purified human FH to the FHbp mutants by ELISA. In this assay, the previously described R41S mutant [22] bound less than 200-fold as much FH as the wild-type FHbp (Fig 3A). Three of the mutants identified from the library, E92K, S223R and H248L, displayed even lower FH binding than the R41S mutant (Fig 3B). The other two mutants, Q38R and R130G, had significantly decreased FH binding compared with the wild-type FHbp, but had more FH binding than the R41S mutant.

**Fig 4:**
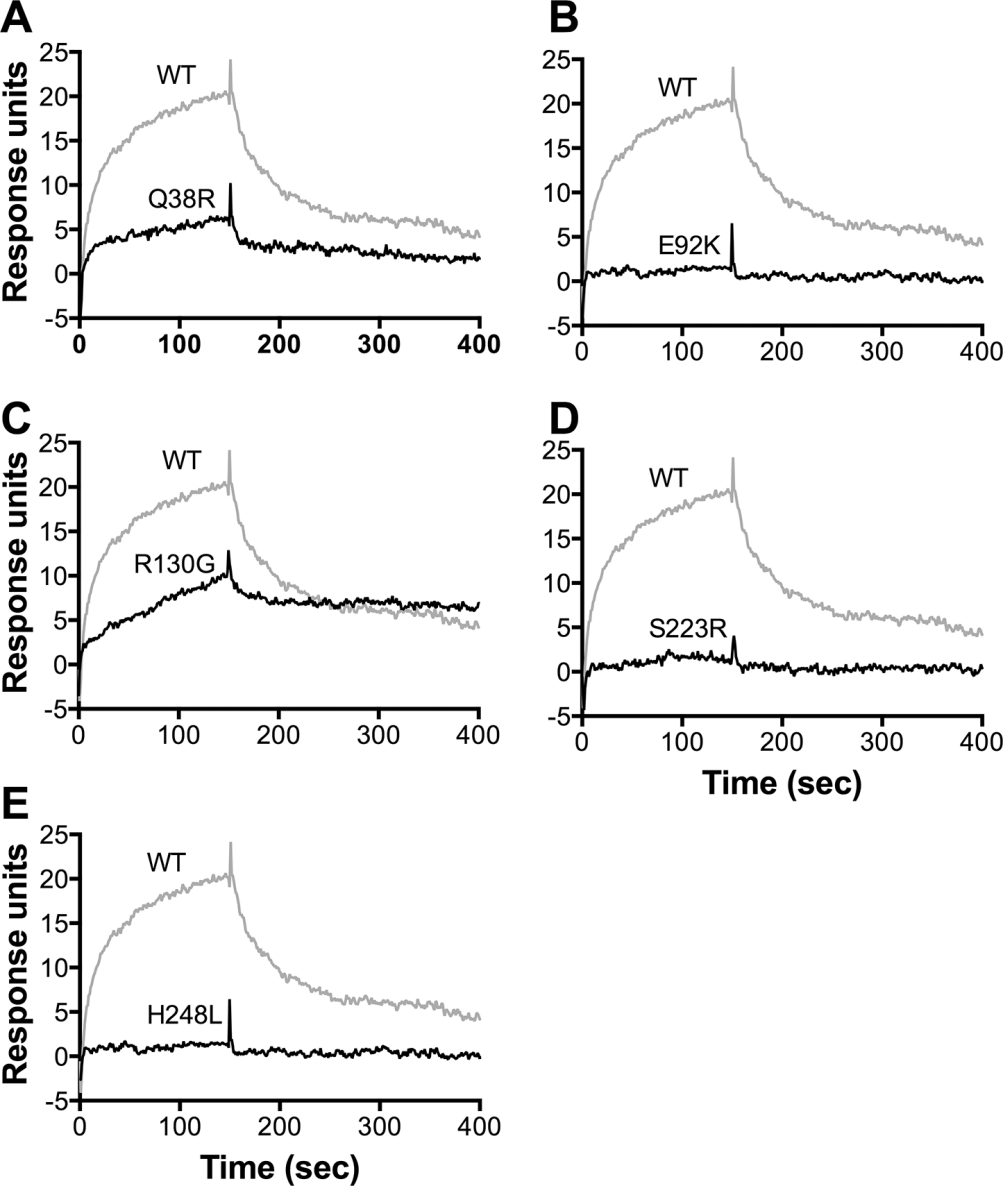
Binding of FHbp to immobilized human FH by surface plasmon resonance. Purified human FH (1500 response units) was coupled to the biosensor chip. Different concentrations (3 to 316 nM) of purified, recombinant FHbp were used to determine equilibrium dissociation constants (**Table 1**). **A.** Data for injection of 100 nM of wild-type (WT) FHbp (grey line) or Q38R mutant (black line) are shown. **B-E.** Data for E92K, R130G, S223R and H248L mutants are shown with WT FHbp (same data as in **Panel A**). Binding equivalent to two response units was considered as the limit of detection.

We also assessed the effect of the FHbp amino acid substitutions on FH binding in surface plasmon resonance (SPR) experiments using purified human FH coupled to the biosensor chip. FH binding of three mutants, E92K, S223R and H248L, was not detectable by SPR (<2 response units at the highest FHbp concentration tested, 100 nM; (Fig 4B, 4D and 4E). The remaining two mutants, R130G and Q38R, bound FH with equilibrium dissociation constants (*K*_*D*_) of 65 and 244 nM, respectively, compared with the control wild-type FHbp, which had a *K*_*D*_ of 17 nM (Fig 4A and 4C and Table 2). Thus, the three FHbp mutants that had the lowest FH binding by ELISA also showed no detectable FH binding by SPR.

**Table 2:** FH binding and stability data for FHbp mutants.

FHbp^a^	FH binding		Thermal Stability	
	Fold-decrease^b^	*K*_*D*_ (nM)^c^	*T*_*m*_1 (°C)^d^	*T*_*m*_ 2 (°C)^d^
Wild-type	—-	17	69.4	84.9
R41S^e^	238	No binding	67.9	84.7
Q38R	158	244	71.0	85.2
E92K	>250	No binding	54.2	84.9
R130G	50	65	61.8	85.0
S223R	>250	No binding	69.5	86.4
H248L	>250	No binding	68.3	85.0

^a^ Numbering of mutants is based on the mature sequence of FHbp ID 1 (http://pubmlst.org/neisseria/fHbp) with the lipidated cysteine as residue number 1.

^b^ Fold-decrease in FH binding relative to wild-type FHbp by ELISA (OD_405_ = 1.5); the mutants I114T, I134V, H138L, K199E, S211T and G236C (not shown) had <10-fold decreases in FH binding.

^c^ *K*_*D*_, equilibrium dissociation constant measured by surface plasmon resonance

^d^ Transition midpoints for amino-terminal domain (*T*_*m*_ *1*) and carboxyl-terminal domain (*T*_*m*_ *2*) by scanning calorimetry.

^e^ Previously described R41S mutant [22] was used as a control in the present study

### Conformational stability of FHbp mutants

All of the FHbp mutants bound anti-FHbp mAb JAR 5 [40], which binds to a relatively stable but discontinuous epitope in the amino-terminal domain of FHbp [41], similarly to the wild-type FHbp (Fig 3C). Binding of two other anti-FHbp mAbs, JAR 4 [40, 42] and mAb502 [30, 43], which recognize conformational epitopes in each of the two structural domains of FHbp, to the mutants also was indistinguishable from the wild-type (data not shown). Thus, despite having significantly decreased binding of FH, all of the FHbp mutants had preserved epitopes in three distinct regions of the protein.

**Fig 5:**
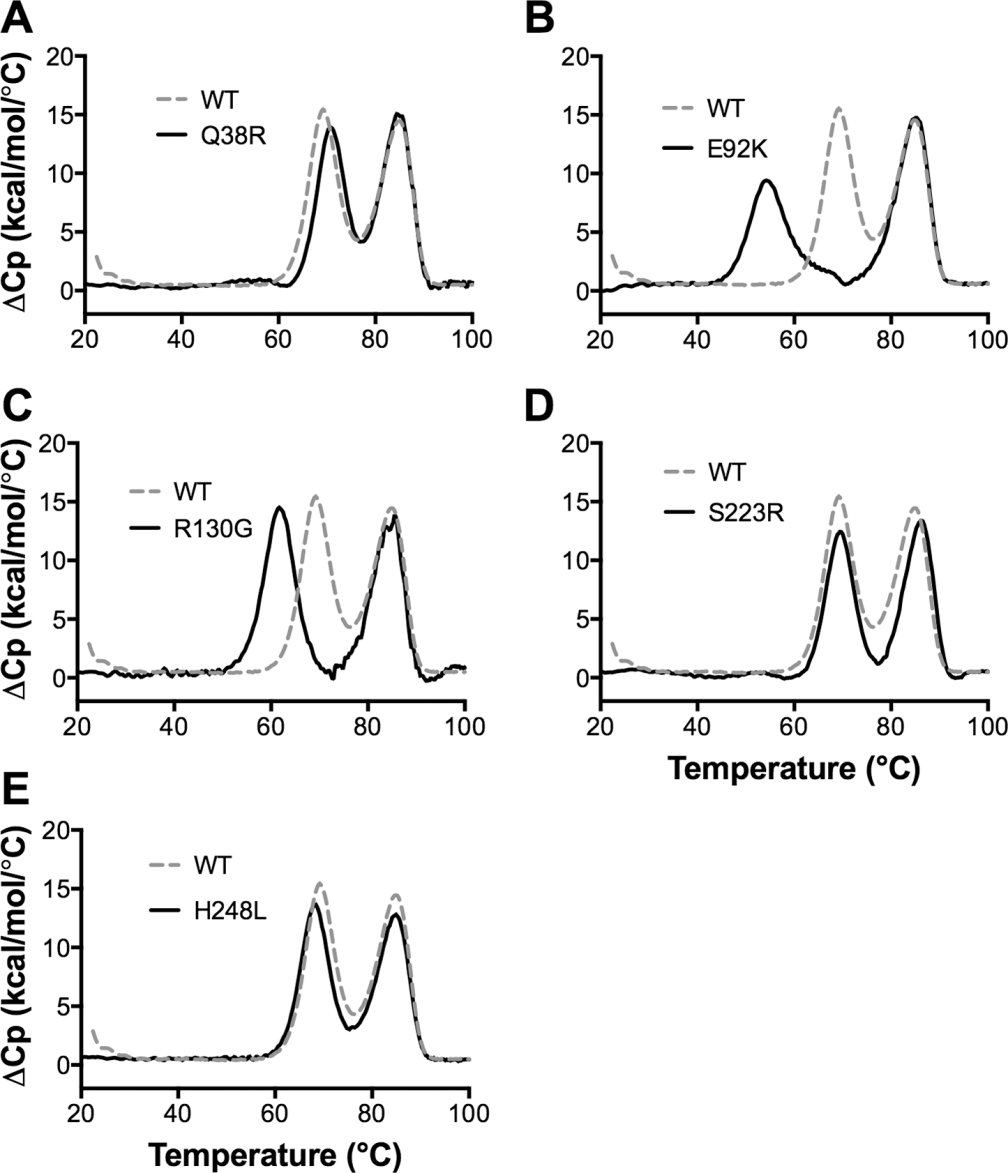
Thermal stability of FHbp ID 1 mutants measured by differential scanning calorimetry. **A.** Wild-type (WT) FHbp ID 1 (dashed grey line) and Q38R mutant (solid black line). **B-E.** E92K, R130G, S223R and H248L mutants (solid black lines), each shown compared to the same data for the WT ID 1 as in Panel A. FHbp (0.5 mg/ml in PBS) was heated at a scan rate of 60°C/h and the excess heat capacity (ΔCp) was measured. Reference buffer data were subtracted and the data were normalized based on the molecular weight of FHbp (27.7 kDa). The lower and higher temperature transitions corresponded with the unfolding of the N- and C-terminal domains [44].

To test the structural integrity of the FHbp mutants, we measured their thermal stability by differential scanning calorimetry. As previously described [44, 45], the wild-type FHbp unfolded with two transitions, with midpoint (*T*_*m*_) values of 69.5 and 85.4°C (Fig 5A, dashed grey line), which corresponded to unfolding of the amino- and carboxyl-terminal domains, respectively. Three of the FHbp mutants, Q38R, S223R and H248L, had similar stability profiles as the wild-type FHbp (Fig 5A, 5D and 5E). The E92K and R130G mutants had decreased stability for the amino-terminal domain (Fig 5B and 5C, solid black lines), compared with the wild-type FHbp. Based on the results of FH binding and thermal stability experiments, the FHbp mutants S223R and H248L appeared to be the most promising vaccine candidates.

### Protective antibody responses of mice to mutant FHbp vaccines

**Fig 6:**
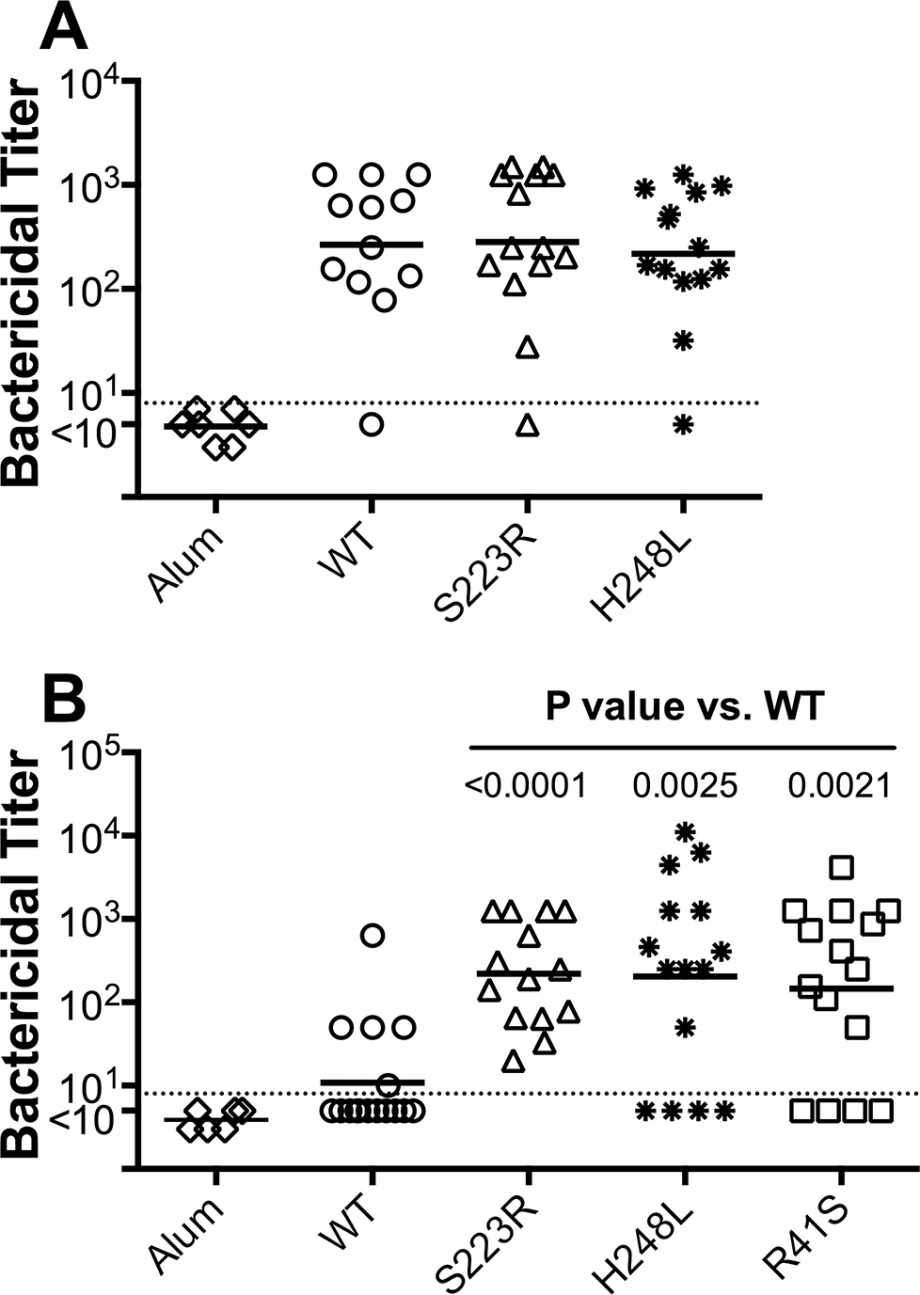
Serum bactericidal antibody responses of mice to mutant FHbp vaccines. **A.** Control mice immunized with FHbp mutant vaccines. Each symbol represents the serum bactericidal antibody titer of an individual mouse and the horizontal lines represent the geometric mean titer (GMT) of each vaccine group. There were no statistically significant pairwise differences between the WT FHbp and either of the FHbp mutants (P≥ 0.62). **B.** Human FH transgenic mice immunized with FHbp mutant vaccines. The GMT for the R41S mutant was 13-fold higher than for the wild-type FHbp, 20-fold higher for S223R, and 19-fold higher for H248L. The P values for Mann-Whitney tests comparing each mutant group with the WT group are shown.

To assess the ability of the FHbp mutant vaccines to elicit protective serum antibodies, we immunized groups of CD-1 mice with two doses of each of the two FHbp mutants with very low FH binding and preserved thermal stability, S223R and H248L, or a control wild-type FHbp vaccine. The FHbp mutations that decreased binding of human FH were not expected to affect the magnitudes of the protective antibody responses of mice, whose FH does not bind to FHbp, unless the mutation adversely affected important epitopes. Accordingly, we tested the protective antibody responses against serogroup B strain H44/76 [36], which expresses an FHbp sequence that is an identical match to the vaccine. The bactericidal geometric mean titer (GMT) elicited in mice given the wild-type FHbp vaccine was 266, and the GMTs of mice given the FHbp mutant vaccines ranged from 153 (E92K) to 283 (Fig 6A). There were no significant pairwise differences between the responses of mice immunized with the FHbp mutants compared with the wild-type vaccine. Thus, in mice whose FH did not bind to the FHbp vaccine, the mutations that decreased FH binding did not impair FHbp immunogenicity.

To test the hypothesis that mutant FHbp vaccines with decreased binding of human FH have enhanced protective antibody responses in the presence of human FH, we immunized human FH transgenic BALB/c mice with three doses of FHbp vaccines. As a control in the transgenic mouse immunogenicity study, we included the R41S mutant, which in a previous study elicited increased protective antibody responses in human FH transgenic mice [22]. When measured against serogroup B strain H44/76, only four of fifteen mice vaccinated with the wild-type FHbp had titers >10, whereas for the FHbp mutants 11 of 15 (R41S and H248L) or 14 of 14 (S223R) had titers >10. Further, all three of the mutant FHbp vaccines tested elicited higher protective responses than the control wild-type vaccine (P≤0.0025; Fig 6B). The GMT for the R41S mutant was 13-fold higher than that for the wild-type (146 vs.11; P = 0.002), the S223R mutant was 20-fold higher (222 vs. 11; P = 0.0001) and the H248L mutant was 19-fold higher (204 vs. 11; P = 0.003). Although the GMTs elicited by the two new mutants were higher than that of the R41S mutant, the respective pair-wise differences were not statistically significant (P>0.70).

## Discussion

Human FH binds to meningococcal FHbp and decreases the protective antibody responses to vaccines containing FHbp as compared to mouse FH, which does not bind to FHbp [22, 38]. Serum antibodies from control mice immunized with a vaccine containing FHbp elicited higher bactericidal activity and better inhibited FH binding to FHbp compared to antibodies from human FH transgenic mice, which unexpectedly enhanced FH binding [38]. Our recent study of the serum antibody responses of infant rhesus macaques to a vaccine containing FHbp was consistent with these findings. Macaques with low binding of FH to FHbp had higher protective antibody responses than macaques with high binding. However, both groups of animals developed antibodies that enhanced binding of FH to FHbp, which suggested that even low binding of FH could decrease the antibody responses to FHbp vaccines [17]. Collectively, these data suggest that FHbp mutants with very low FH binding will be more effective vaccine antigens in humans than wild-type FHbp that binds FH.

Several previous studies have identified FHbp mutants with decreased FH binding as candidates for improved meningococcal serogroup B vaccines [22, 25–28]. Further, in one of the studies involving two FHbp mutants with low FH binding, the mutant with lower FH binding elicited higher protective antibody responses in human FH transgenic mice [26]. In the present study, we developed a novel, random mutant library approach to identify meningococcal FHbp antigens with very low binding of human FH. To discriminate among mutants with low FH binding, we adapted our previously described FH ELISA [22] to employ concentrations of purified human FH as high as 200 μg/ml, which approximates those in human serum [22]. Using these approaches, we identified several new FHbp mutants that had substantially lower FH binding than the previously described R41S mutant, and with more than 250-fold decreased binding of human FH compared with wild-type FHbp.

Two important requirements for an ideal non-FH binding FHbp mutant are that the mutation: 1) not perturb epitopes recognized by protective antibodies (as assessed in control mice); and 2) increase the protective antibody responses in the presence of human FH (as assessed in human FH transgenic mice). In previous studies, despite a slight decrease in the protective antibody responses of control mice to the R41S mutant compared with the wild-type FHbp, we observed an increase in the responses of human FH transgenic mice to the R41S mutant [22, 24]. In one of these studies that used a native outer membrane vesicle vaccine platform, the R41S mutant elicited four-fold lower protective antibody responses in control mice and 19-fold higher responses in human FH transgenic mice. Therefore, we reasoned that substitutions to decrease FH binding that did not decrease antibody responses in control mice would elicit even higher responses than the FHbp R41S mutant vaccine in human FH transgenic mice.

The new mutants that we identified had very low FH binding, retained epitopes recognized by multiple anti-FHbp mAbs and retained immunogenicity in control mice. In human FH transgenic mice, the mutants elicited protective antibody responses that were higher than the previously described R41S mutant [22] and as much as 20-fold higher than the wild-type FHbp. Since the pairwise differences between the new mutants and the R41S mutant were not statistically significant, larger groups of transgenic mice would be needed to determine whether the new mutants are superior. In either case, the new mutants might be valuable in producing second-generation double mutants with even lower FH binding, or in combining two different single mutants into a FHbp vaccine that retains all of the native epitopes.

One limitation of the library approach for discovery of FHbp mutants is that induction of FHbp expression appeared to be moderately toxic to *E*. *coli*. As a result, the mutant FHbp clones could not be retested directly by flow cytometry. Since the structures of FHbp alone [39] and in a complex with an FH fragment [23] had been determined, we used this structural information to prioritize single amino acid positions that were in or near the FH binding site. For other microbial antigens that did not display toxicity in *E*. *coli*, mutants with low binding of the cognate host protein could be confirmed by flow cytometry without knowledge of the amino acid residues in the binding site. This modified approach would streamline the process so that the steps of sequencing, site-specific mutagenesis and protein production would be applied only those clones confirmed to have low binding to the host protein.

One advantage of the library approach is that various amino acid substitutions can be identified at any given position. For any codon, an average of 6 possible missense mutations can be generated from a single nucleotide change. This potential for diverse substitutions is important, since a previous study found that alanine substitutions at positions S223 or H248 had no or only moderate decreases in binding of FH (1.2-fold and 9.6-fold, respectively) [27]. In contrast, in the present study the S223R and H248L substitutions had >250-fold decreases in binding of FH, yet retained important epitopes as determined by binding of anti-FHbp mAbs and preserved immunogenicity in control mice.

In summary, two previous studies have shown that human FH decreases FHbp vaccine immunogenicity [22, 38]. Further, studies by several groups have shown that the decrease in immunogenicity can be overcome by introduction of FHbp mutations that decrease binding of FH [22, 24, 26–28]. Many of these previous studies as well as the present study were performed using transgenic mice that expressed serum human FH at concentrations similar to the range found in humans [22, 24, 26], and collectively demonstrate that mutations in FHbp antigens can augment the protective antibody responses by at least 20-fold. We anticipate that the responses of humans would be increased similarly, which would translate into more efficacious meningococcal serogroup B vaccines for humans. The library-based approach for discovery of mutants potentially can be applied to other microbial antigens that interact with host proteins, including those for which a three-dimensional structure of the microbial antigen in a complex with the host protein is not available.
